# Atmospheric Precipitations, Hailstone and Rainwater, as a Novel Source of *Streptomyces* Producing Bioactive Natural Products

**DOI:** 10.3389/fmicb.2018.00773

**Published:** 2018-04-23

**Authors:** Aida Sarmiento-Vizcaíno, Julia Espadas, Jesús Martín, Alfredo F. Braña, Fernando Reyes, Luis A. García, Gloria Blanco

**Affiliations:** ^1^Departamento de Biología Funcional, Área de Microbiología, e Instituto Universitario de Oncología del Principado de Asturias, Universidad de Oviedo, Oviedo, Spain; ^2^Fundación MEDINA, Centro de Excelencia en Investigación de Medicamentos Innovadores en Andalucía, Parque Tecnológico de Ciencias de la Salud, Granada, Spain; ^3^Departamento de Ingeniería Química y Tecnología del Medio Ambiente, Área de Ingeniería Química, Universidad de Oviedo, Oviedo, Spain

**Keywords:** hailstorm, bioaerosols, *Streptomyces*, antibiotic, antimicrobial, antitumor

## Abstract

A cultivation-dependent approach revealed that highly diverse populations of *Streptomyces* were present in atmospheric precipitations from a hailstorm event sampled in February 2016 in the Cantabrian Sea coast, North of Spain. A total of 29 bioactive *Streptomyces* strains isolated from small samples of hailstone and rainwater, collected from this hailstorm event, were studied here. Taxonomic identification by 16S rRNA sequencing revealed more than 20 different *Streptomyces* species, with their closest homologs displaying mainly oceanic but also terrestrial origins. Backward trajectory analysis revealed that the air-mass sources of the hailstorm event, with North Western winds, were originated in the Arctic Ocean (West Greenland and North Iceland) and Canada (Labrador), depending on the altitude. After traveling across the North Atlantic Ocean during 4 days the air mass reached Europe and precipitated as hailstone and rain water at the sampling place in Spain. The finding of *Streptomyces* species able to survive and disperse through the atmosphere increases our knowledge of the biogeography of genus *Streptomyces* on Earth, and reinforces our previous dispersion model, suggesting a generalized feature for the genus which could have been essential in his evolution. This unique atmospheric-derived *Streptomyces* collection was screened for production of bioactive secondary metabolites. Analyses of isolates ethyl acetate extracts by LC-UV-MS and further database comparison revealed an extraordinary diversity of bioactive natural products. One hundred molecules were identified, mostly displaying contrasted antibiotic and antitumor/cytotoxic activities, but also antiparasitic, antiviral, anti-inflammatory, neuroprotector, and insecticide properties. More interestingly, 38 molecules not identified in natural products databases might represent new natural products. Our results revealed for the first time an extraordinary diversity of *Streptomyc*es species in the atmosphere able to produce an extraordinary repertoire of bioactive molecules, thus providing a very promising source for the discovery of novel pharmaceutical natural products.

## Introduction

Natural products are essential to human health and constitute a primary resource in biomedicine and biotechnology. *Streptomyces* species (Phylum *Actinobacteria*) are the most prolific source of bioactive natural products with pharmaceutical activities. New trends in the discovery of novel drugs, such antibiotics and antitumor compounds, are focused on the search of producing microorganisms from unexplored habitats (Subramani and Aalbersberg, 2013; Behie et al., 2016; Maciejewska et al., 2016; Law et al., 2017).

Although *Streptomyces* species have been traditionally considered as soil bacteria, in the last decades became evident their presence and wide distribution in oceanic ecosystems and associated to diverse marine organisms. Previous work in the North Atlantic region, Cantabrian Sea (Bay of Biscay), Northern Spain, revealed the presence of a great number of *Streptomyces* strains in intertidal seaweeds, and deep-sea coral reef invertebrates at the Aviles Canyon. New natural products with antimicrobial and cytotoxic activities against tumor cell lines were recently discovered in this submarine Canyon (Braña et al., 2015, 2017a,b; Sarmiento-Vizcaíno et al., 2015, 2016, 2017a,b).

Besides Earth and oceans, there is increasing evidence of the presence of *Streptomyces* strains in the atmosphere. In culture-dependent approaches, *Streptomyces* strains were isolated from cloud water at Puy de Dôme, Southern France (Amato et al., 2007) and repeatedly isolated from atmospheric precipitations such as rainwater, hailstone and snow in the Cantabrian region, during 2013–2014 (Braña et al., 2015; Sarmiento-Vizcaíno et al., 2016). These included three ubiquitous species, *Streptomyces albidoflavus, Streptomyces cyaneofuscatus*, and *Streptomyces carnosus*, previously isolated from terrestrial and oceanic environments (Braña et al., 2015; Sarmiento-Vizcaíno et al., 2016).

Following this line of evidence, we have previously proposed an atmospheric dispersion model, which follows the Earth hydrological cycle, to explain the biogeography and distribution among terrestrial, marine and atmospheric environments of *Streptomyces* species (Sarmiento-Vizcaíno et al., 2016). According to this hypothetical cycle, oceanic bioaerosols-forming clouds contribute to streptomycetes dissemination from marine ecosystems to the atmosphere, where they undergo long distances transport by winds and finally fall down to inland and oceanic ecosystems by precipitation.

Clouds have been defined as atmospheric air masses with water condensed in ice crystals or liquid state (Amato et al., 2007). They are considered as low-temperature “aquatic” environments which contribute to transport and aerial connection between Earth ecosystems (Amato et al., 2007), having been considered as possible atmospheric oases for microorganisms (Amato et al., 2017). Recent studies at the Puy de Dôme Mountain revealed that some microorganisms are even metabolically active in clouds (Amato et al., 2017). Rainwater droplets can coalesce into hailstones which circulate inside storm clouds following unpredictable pathways (Amato et al., 2017). Biogeochemical studies on hailstones indicate that storm clouds can be considered among the most extreme habitats for microbial life on Earth. (Šantl-Temkiv et al., 2013).

Here is reported the exploration of the phylogenetic and biosynthetic diversity of atmospheric-derived *Streptomyces* strains collected from a storm cloud in Northern Spain, using hailstone and rainwater precipitations as natural sampling sources. This work constitutes the first large insight into the *Streptomyces* diversity existing in hailstone and rainwater and the natural compounds produced. Meteorological analyses addressed to estimate the air mass sources and trajectories support our previous *Streptomyces* dispersion cycle model.

## Materials and methods

### Sampling of atmospheric precipitations (hailstone and rain water)

**Figure 1:**
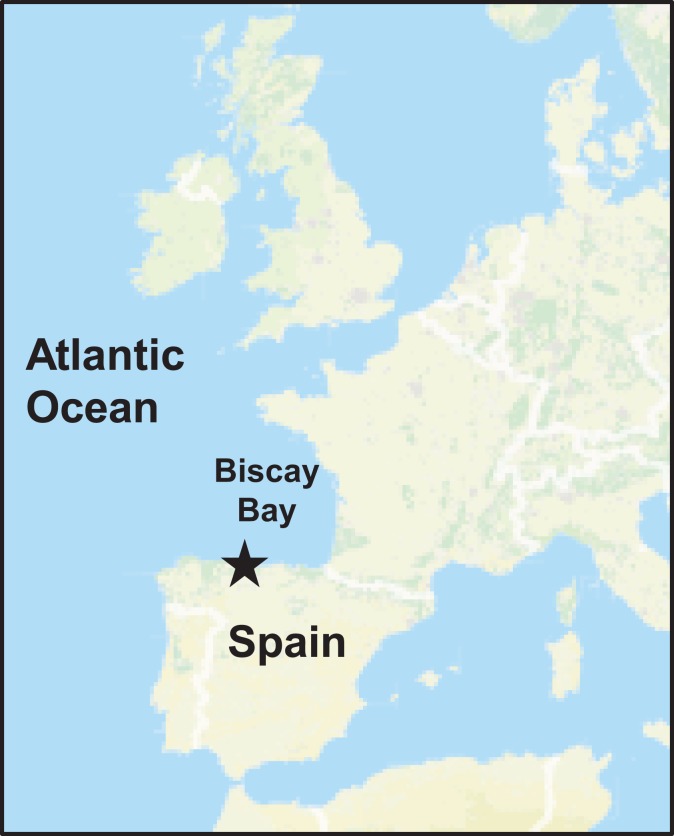
Sampling location. Overview of the Western European Seas (Atlantic Ocean). *Star* indicates the sampling location at Gijón, North of Spain (Iberian Peninsula).

Cloud precipitations samples, such as hailstone and rainwater, were taken during a thunderstorm discharge over the coastal location of Gijón (Asturias) in the afternoon of 14th February 2016 at 16.00 h. Gijón (43°32′ N, 5°39′ W) is located in the North of Spain (Bay of Biscay, Figure 1). The prevailing wind direction during this storm event in this area was Northwestern.

Hailstone and rainwater samples from this storm event were collected in sterile recipients, while they were falling on the ground, in a terrace in front of the sea at about 30 m above sea level. Samples were stored at −20°C (hailstone) and 4°C (rain water) until immediately processing as has been reported (Braña et al., 2015).

### Air mass backward trajectories analysis

In order to investigate the long-range transport journey of air masses that originated the precipitation event, backward trajectories were obtained using the HYSPLIT model (Hybrid Single Particle Lagrangian Integrated Trajectory) obtained from the Global Data Assimilation System of National Oceanic and Atmospheric Administration, USA (Stein et al., 2015). To track the transport pathways of air masses, 5-day backward trajectories (commonly used in bioaerosol studies) were generated using the NOAA model (http://ready.arl.noaa.gov/hypub-bin/trajtype.pl?runtype=archive) to determine the origin of a given air parcel. The sampling location for this study was used as the backward trajectory start point with altitudes of 30, 1,000, and 3,000 m, respectively above the ground level to estimate the accurate trajectories of atmospheric air masses.

### Isolation of *Streptomyces* strains and culture media

Atmospheric samples were inoculated on selective agar media prepared with cycloheximide (80 μg ml^−1^) as antifungal and nalidixic acid (20 μg ml^−1^) as anti-Gram negative bacteria, using MOPS BLEB 1/6 (Oxoid) basal medium as previously reported (Sarmiento-Vizcaíno et al., 2016). For selection, two different media either prepared with distilled water or with a supplement of 3.5% NaCl were used. After 2–3 weeks of incubation at 28°C, growing colonies were selected based on different morphological features and pigment production on R5A agar plates. Isolates obtained in pure cultures were conserved in 20% glycerol at −20°C, and at −70°C. For addressing halotolerance studies, MOPS BLEB 1/6 (Oxoid) was used as the basal medium, adding NaCl at of 0, 3.5, 7.0, and 10.5% (w/v) final concentrations. For secondary metabolite production streptomycetes isolates were cultured on R5A medium as previously described (Braña et al., 2015).

### Antimicrobial bioassays

Agar diffusion methods were used to determine antimicrobial activities. Antibiotic production was assessed using the following indicator microorganisms: the Gram-positive bacteria *Micrococcus luteus* ATCC 14452 and *Streptomyces* 85E ATCC 55824 (Shanbhag et al., 2015), the Gram-negative *Escherichia coli* ESS, and the yeast *Saccharomyces cerevisiae* var. *carlsbergensis*. Analyses were performed in TSA1/2 (Merck) against bacteria and in Sabouraud 1/2 (Pronadisa) against yeast. Bioassays were carried out both with agar plugs (7 mm diameter) and in parallel with different ethyl acetate extracts obtained from solid cultures of the isolates.

### 16S RNA phylogenetic analysis

For phylogenetic analysis of the strains based on 16S rRNA sequences, DNA was extracted with a microbial isolation kit (Ultra Clean, MoBio Laboratories, Inc.) and standard methods were used for checking the purity (Russell and Sambrook, 2001). Partial 16S rRNA gene sequences of the bacterial strains were obtained by using the 616V (forward) and 699R (reverse) primers (Arahal et al., 2008) in PCR amplification as previously described (Braña et al., 2015). Once obtained the nucleotide sequences were compared to sequences in databases using the BLAST program (Basic Local Alignment Search Tool) against the NCBI (National Centre for Biotechnology Information). The nucleotide sequences were submitted and deposited in the EMBL sequence database. Phylogenetic analysis of the strains based on 16S rRNA sequences was carried out as previously reported (Sarmiento-Vizcaíno et al., 2017a).

### Chromatographic analysis

Plugs of R5A plates (about 7 ml) were extracted using ethyl acetate in neutral and acidic (with 1% formic acid) conditions. After evaporation, the organic fraction residue was redissolved in 100 μL of a mixture of DMSO and methanol (50:50). The analysis of the samples were performed by reversed phase liquid chromatography as has been described (Braña et al., 2014; Sarmiento-Vizcaíno et al., 2016).

### Identification of compounds by LC-UV-Vis and LC-UV-HRMS analyses

Samples were first analyzed and evaluated using an in-house HPLC-UV-Vis database. LC-UV-HRMS analyses were carried out as has been described (Pérez-Victoria et al., 2016) and major peaks in each chromatogram were searched against the MEDINA's internal database and also against the Dictionary of Natural Products (DNP) (Chapman and Hall/CRC, 2015).

## Results

### Backward transport trajectories

**Figure 2:**
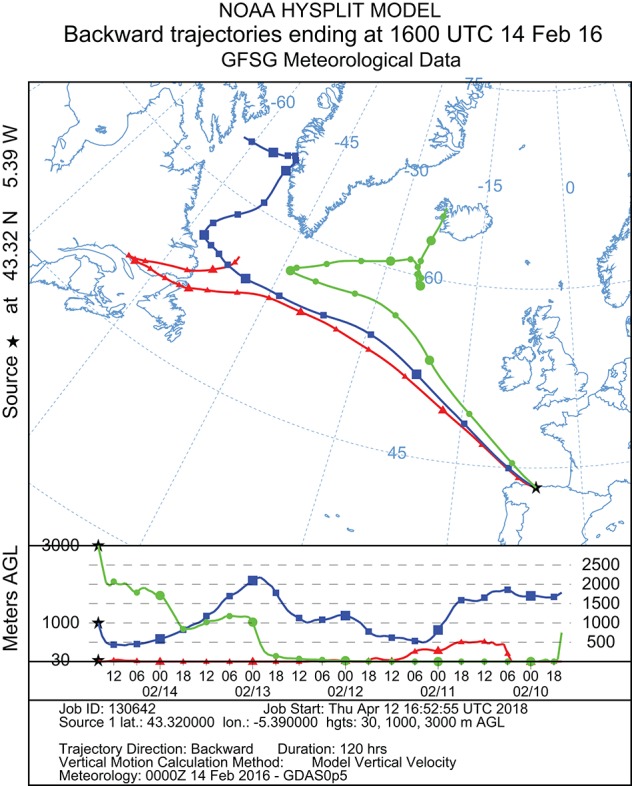
Five-day backward trajectories of air masses generating the storm that arrived at Gijón (North Spain) on February 14, 2016, at 16.00, calculated with the NOAA Hysplit Model with three different transects with different arriving height: red = 30 m; blue = 1,000 m; green = 3,000 m. Sampling time and place are indicated by the black asterisk.

To estimate the sources of the air masses that caused the precipitation event in the Cantabrian Sea coast, 120 h backward trajectories were determined at three different arriving heights (30, 1,000, and 3,000 m). As shown in Figure 2, the results of the NOAA meteorological analysis indicated three different routes followed by the air layers depending on the altitude. The air-mass at 30 m altitude originate from Newfoundland and Labrador (Canada); the one at 1,000 m came from the Davies Strait in the Arctic Ocean, between North Canada and West Greenland; the air layer at 3,000 m originated at Northwest Iceland. All air-masses at different altitudes crossed the Atlantic Ocean, and after 4 days reached the Iberian Peninsula precipitating as hailstones and rainwater in the North of Spain, were samples were collected. In addition, possible mixing events were detected between different air layers at different altitudes meanwhile traveling across the Atlantic Ocean (Figure 2). Thus, the estimated backward trajectories mainly revealed an oceanic route, involving both the Arctic and Atlantic Oceans, but there was also a terrestrial route from continental America.

### Isolation of bioactive *Streptomyces* from a hailstorm event

A cultivation-dependent approach revealed in atmospheric precipitations the presence of highly diverse *Streptomyces* populations by using a sample of hailstone (300 ml of unfrozen hailstones) and a sample of 25 ml of rainwater. Only strains cultivable at 28°C and atmospheric pressure were recovered on selective agar plates, prepared either with 3.5% NaCl (to simulate the salt content of the Cantabrian Sea water) or without salt. The percentage of streptomycete colonies recovered on selective medium without salt was higher, approximately 66% from hailstone and 56% from rainwater, than on saline medium.

Among a total of 136 streptomycete colonies isolated on selection plates (92 colonies from hailstone and 44 from rainwater), 45 morphologically different isolates from hailstone and 14 from rainwater samples were selected after the dereplication process. None of these isolates required NaCl for growth. An important feature observed in all isolates, with a single exception, was their halotolerance. Most isolates tolerate around 7% NaCl, which doubles the salt concentration in the Cantabrian Sea (3.5% average). This is in agreement with previous studies of NaCl tolerance for the genus *Streptomyces* in which it was estimated that around 50% of the species tolerate up to 7% NaCl (Tresner et al., 1968).

All isolates were initially tested for antimicrobial activity using agar diffusion assays. Further 29 different bioactive strains, displaying diverse antimicrobial activities, were selected for this study (Table 1). Most of the isolates displayed antibiotic activities against *M. luteus* and *Streptomyces* 85E (Gram-positive bacteria), and also against the yeast *S. cerevisiae*; whereas only three strains were active against the *E. coli* ESS (Gram-negative).

**Table 1:** Antibiotic activities of *Streptomyces* cultures (agar plugs) against Gram-positive, Gram-negative bacteria, and yeasts.

**Strain**	***M. luteus***	***Streptomyces* 85E**	***E. coli***	***S. cerevisiae***
A-185	20	–	–	20
A-186	22	–	–	–
A-189	–	–	–	15
A-191	11	26	–	10
A-192	–	–	18^*^	–
A-193	–	13	–	–
A-196	–	12	–	–
A-197	20	21	18	–
A-198	–	–	–	19
A-201	12	10	–	–
A-202	11	12	–	–
A-203	13	17	–	10
A-204	27	28	–	–
A-206	14	22	19	13
A-208	16	–	–	–
A-209	19	–	–	–
A-210	13	11	–	–
A-211	13	13	–	–
A-214	11	36	–	–
A-215	16	–	–	–
A-217	26	33	–	–
A-221	16	33	14	14
A-222	–	–	–	–
A-225	19	15	–	–
A-226	14	–	–	–
A-227	–	11	–	–
A-228	–	10	–	16
A-229	13	–	–	11
A-230	–	–	–	15
A-231	11	–	–	11

*Activities were measured as the zones of complete inhibition (diameters in mm). Bioassays with ethyl acetate extracts in acid and neutral conditions were also performed in parallel. The asterisk indicates that the activity was detected in the extract*.

### Taxonomic identification of isolates

**Figure 3:**
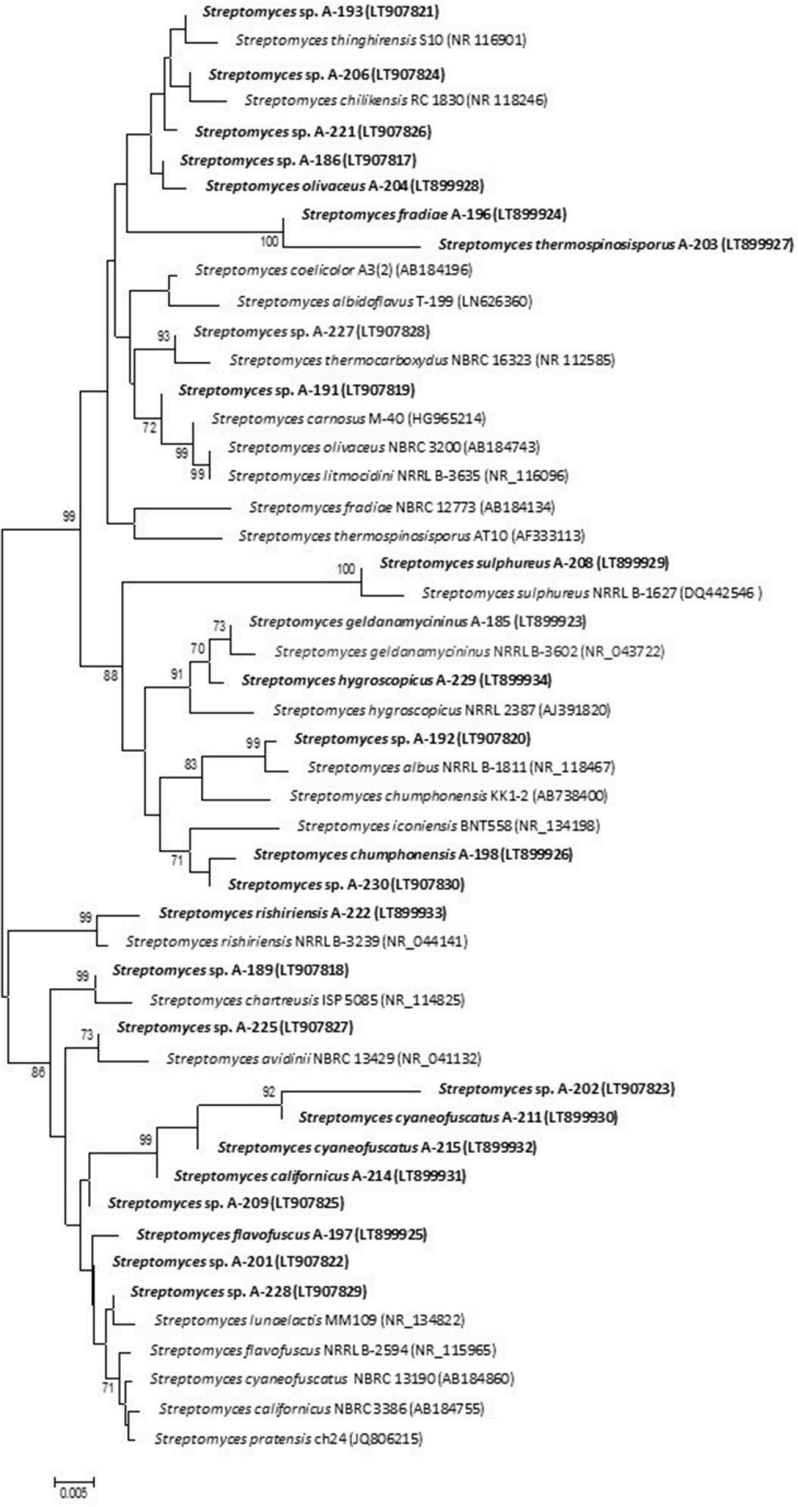
Neighbor-joining phylogenetic tree generated by distance matrix analysis of 16S rDNA sequences from atmospheric *Streptomyces* strains (highlighted) and nearest phylogenetic relatives. The numbers on branch nodes indicate bootstrap values (1,000 resamplings; only values >70% are shown). Bar represents 0.5% sequence divergence.

For taxonomic identifications of the atmospheric-derived bioactive strains, we sequenced fragments of their 16S rDNA and deposited the nucleotide sequences in the EMBL database. Table 2 displays the accession numbers of the strains. Based on 16S rRNA gene alignments, phylogenetic analyses clearly demonstrated that all 29 isolates belonged to the *Streptomyces* genus, since all of them shared 99–100% identity with previously known *Streptomyces* species. The relationship between the atmospheric isolates and their closest phylogenetic relatives with indication of their isolation site is shown in Table 2. A phylogenetic tree was built to display the diversity among atmospheric isolates and assess their phylogenetic relationship (Figure 3).

**Table 2:** Phylogenetic diversity of atmospheric-derived bioactive *Streptomyces* isolates.

**Strain**	**Source**	**EMBL A. N**.	**NaCl %**	**Closest homolog**	**A. N**.	**% Homology (bp)**	**Isolation source (reference)**
*Streptomyces geldanamycininus* A-185	Rain water	LT899923	<3.5	*Streptomyces geldanamycininus* NRRL B-3602	NR_043722	99.9 (727/728)	Soil (Goodfellow et al., 2007)
*Streptomyces* sp. A-186	Rain water	LT907817	7	*Streptomyces chilikensis* RC 1830*	NR_118246	100 (684/684)	Brackish water sediment Chilika Lake (India) (Ray et al., 2013)
*Streptomyces* sp. A-189	Rain water	LT907818	3.5	*Streptomyces chartreusis* ISP 5085*	NR_114825	100 (769/769)	African soil (Leach et al., 1953)
*Streptomyces* sp. A-191	Rain water	LT907819	7	*Streptomyces litmocidini* NRRL B-3635*	NR_116096	99.7 (768/770)	Soil (Dodzin et al., 1998)
*Streptomyces* sp. A-192	Rain water	LT907820	7	*Streptomyces albus* NRRL B-1811*	NR_118467	99.6 (665/668)	Soil; marine sediment (westsouthern coast Iberian Peninsula) (Schleissner et al., 2011; Labeda et al., 2014)
*Streptomyces* sp. A-193	Rain water	LT907821	7	*Streptomyces thinghirensis* S10*	NR_116901	99.5 (663/666)	Rhizosphere soil of *Vitis vinifer* (Morocco) (Loqman et al., 2009)
*Streptomyces fradiae* A-196	Rain water	LT899924	7	*Streptomyces fradiae* NBRC 12773	AB184134	100 (724/724)	Soil samples (Egypt and Saudi Arabia) (El-Naggar et al., 2016)
*Streptomyces flavofuscus* A-197	Rain water	LT899925	7	*Streptomyces flavofuscus* NRRL B-2594	NR_115965	99.5 (823/827)	Colliery spoil heaps (Czech Republic) (Chronáková et al., 2010)
*Streptomyces chumphonensis* A-198	Rain water	LT899926	7	*Streptomyces chumphonensis* KK1-2	AB738400	98.5 (746/757)	Marine sediment (Tailand) (Phongsopitanun et al., 2014)
*Streptomyces* sp. A-201	Hailstone	LT907822	7	*Streptomyces lunaelactis* MM109*	NR_134822	99.9 (728/729)	Moonmilk deposit from a cave (Belgium) (Maciejewska et al., 2015)
*Streptomyces* sp. A-202	Hailstone	LT907823	7	*Streptomyces pratensis* ch24*	JQ806215	99.4 (855/860)	Grassy fields (Rong et al., 2013)
*Streptomyces thermospinosisporus* A-203	Hailstone	LT899927	7	*Streptomyces thermospinosisporus* AT10	AF333113	99.5 (745/749)	Soil (UK) (Kim and Goodfellow, 2002)
*Streptomyces olivaceus* A-204	Hailstone	LT899928	7	*Streptomyces olivaceus* NBRC 3200	AB184743	99.6 (781/784)	Marine (Yue et al., 2016)
*Streptomyces* sp. A-206	Hailstone	LT907824	7	*Streptomyces chilikensis* RC 1830*	NR_118246	100 (717/717)	Brackish water sediment Chilika Lake (India) (Ray et al., 2013)
*Streptomyces sulphureus* A-208	Hailstone	LT899929	10	*Streptomyces sulphureus* NRRL B-1627	DQ442546	99.7 (621/623)	Marine sediment (China); Submarine Canyon (Spain) (Zhao et al., 2012; Sarmiento-Vizcaíno et al., 2017b)
*Streptomyces* sp. A-209	Hailstone	LT907825	7	*Streptomyces lunaelactis* MM109*	NR_134822	100 (733/733)	Moonmilk deposit from a cave (Belgium) (Maciejewska et al., 2015)
*Streptomyces cyaneofuscatus* A-211	Hailstone	LT899930	7	*Streptomyces cyaneofuscatus* NBRC 13190	AB184860	99.9 (788/789)	Marine, terrestrial and atmospheric (Spain) (Braña et al., 2015)
*Streptomyces californicus* A-214	Hailstone	LT899931	7	*Streptomyces californicus* NBRC 3386	AB184755	100 (789/789)	Saline Soil (USA) (Killham and Firestone, 1984)
*Streptomyces cyaneofuscatus* A-215	Hailstone	LT899932	7	*Streptomyces cyaneofuscatus* NBRC 13190	AB184860	99.2 (763/769)	Marine, terrestrial and atmospheric (Spain) (Braña et al., 2015)
*Streptomyces carnosus* A-217	Hailstone	ND	7	Similar to *Streptomyces carnosus* M-40	HG965214	ND	Marine, terrestrial and atmospheric (Spain) (Braña et al., 2015)
*Streptomyces* sp. A-221	Hailstone	LT907826	7	*Streptomyces chilikensis* RC 1830*	NR_118246	99.6 (746/749)	Brackish water sediment Chilika Lake (India) (Ray et al., 2013)
*Streptomyces rishiriensis* A-222	Hailstone	LT899933	3.5	*Streptomyces rishiriensis* NRRL B-3239	NR_044141	98.7 (602/610)	Soil (Japan) (Matsumoto et al., 1996)
*Streptomyces* sp. A-225	Hailstone	LT907827	3.5	*Streptomyces avidinii* NBRC 13429*	NR_041132	99.5 (605/608)	Marine sediment (India) (Sudha and Masilamani, 2012)
*Streptomyces coelicolor* A-226	Hailstone	ND	7	*Streptomyces coelicolor* A3(2)	AB184196	ND	Soil (UK) (Bentley et al., 2002)
*Streptomyces* sp. A-227	Hailstone	LT907828	7	*Streptomyces thermocarboxydus* NBRC 16323*	NR_112585	99.7 (749/751)	Soil (Kim et al., 1998)
*Streptomyces* sp. A-228	Hailstone	LT907829	3.5	*Streptomyces lunaelactis* MM109^*^	NR_134822	100 (656/656)	Moonmilk deposit from a cave (Belgium) (Maciejewska et al., 2015)
*Streptomyces hygroscopicus* A-229	Hailstone	LT899934	3.5	*Streptomyces hygroscopicus* NRRL 2387	AJ391820	99.8 (803/805)	Soil (Australia) (Jensen, 1931)
*Streptomyces* sp. A-230	Hailstone	LT907830	7	*Streptomyces iconiensis* BNT558^*^	NR_134198	98.9 (730/738)	Salt lake and saltern (Turkey) (Tatar et al., 2014)
*Streptomyces albidoflavus* A-231	Hailstone	ND	7	Similar to *Streptomyces albidoflavus* T-199	LN626360	ND	Marine, terrestrial and atmospheric (Spain) (Sarmiento-Vizcaíno et al., 2016)

*The asterisk indicates that only one species is shown when more than one closest homolog was found. ND, not determined*.

As shown by their identification, strains related to more than 20 different *Streptomyces* species have been isolated from a small sample of hailstone and rainwater (Table 2). This constitutes a significant proportion of the global number of *Streptomyces* species described in our planet so far, estimated in 550–823 (http://www.bacterio.net/streptomyces.html). Among 29 isolates, 15 were identified at species level and were designated with the species name (see Table 2), whereas the rest displayed 16S rDNA gene similarity to more than one species. Unfortunately, just based on these 16S rRNA sequences it is not enough to discriminate among closely related species and further assays are needed to complement the results of 16S rRNA sequence analysis for *Streptomyces* identification at specific level. Among identified isolates, *S. albidoflavus, S. cyaneofuscatus*, and *S. carnosus*, were repeatedly isolated in our geographical area from atmospheric precipitations, marine ecosystems (intertidal seaweeds and deep-sea corals) and terrestrial lichens. Interestingly the model species *Streptomyces coelicolor*, the genetically best known representative of the genus, was here isolated from hailstone; and *Streptomyces albus*, used as heterologous host in many laboratories, was isolated from rain water. Rare or infrequent *Streptomyces* species, here obtained from atmospheric precipitations, were previously isolated from highly diverse environments, mainly from the North Hemisphere (Table 2).

The habitats of known *Streptomyces* species closely related to the atmospheric-derived strains include soils and aquatic environments, such as oceans, lakes, and groundwater. This information was used together with the backward trajectory analysis (see above) to estimate the possible sources of airborne *Streptomyces* reported here.

### Identification of secondary metabolites by metabolite profiling analysis

**Figure 4:**
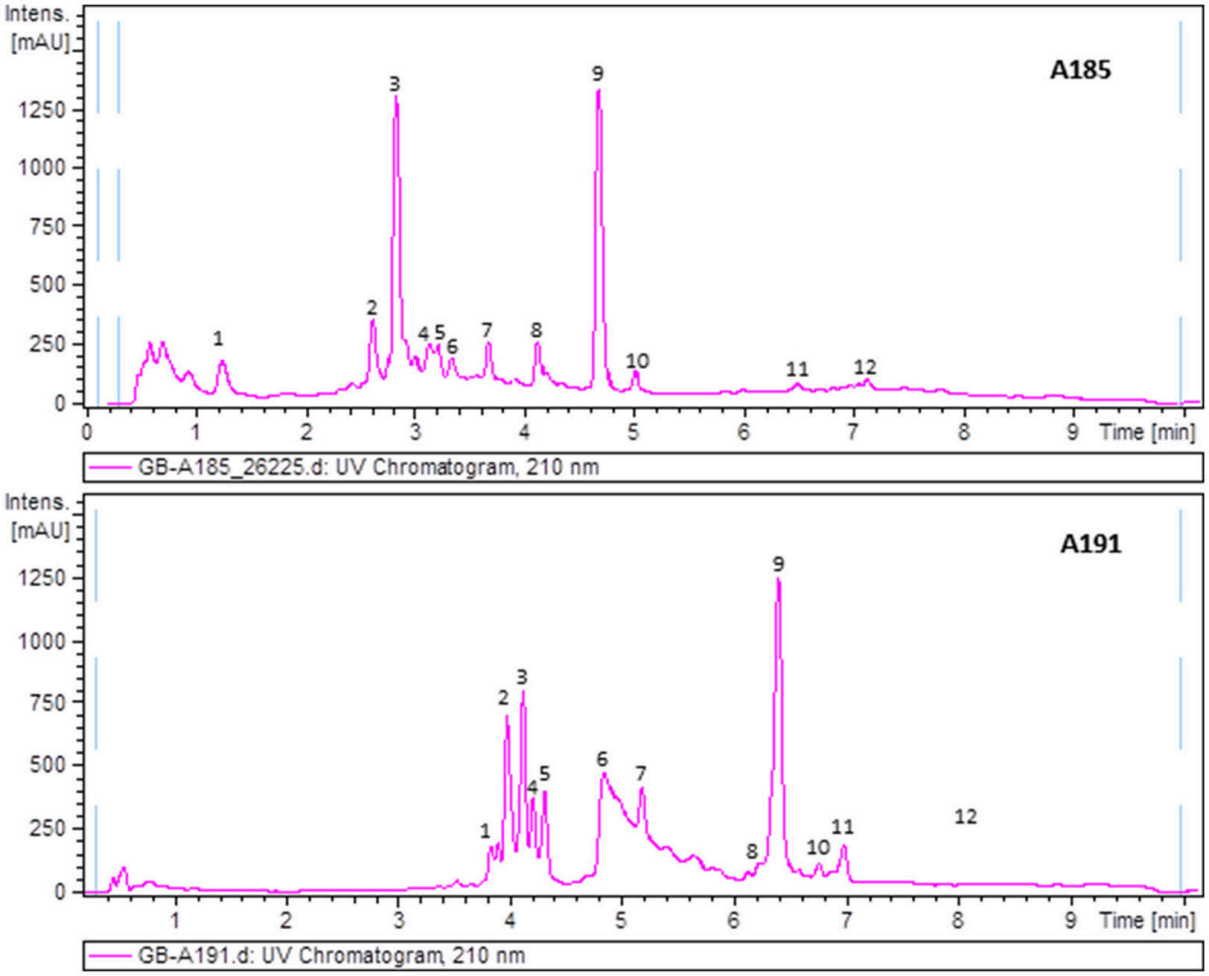
UV_210nm_ chromatogram of samples A185 and A191 with peaks annotated showing components identified by HRMS. Dereplicated components in sample A185: (1) Ketomycin, (2) Thiazinogeldanamycin, (3) 4,5-Dihydrogeldanamycin, (4) Reblastatin, (5) Herbimycin E, (6) 15R-Hydroxygeldanamycin, (7) 6-Demethoxy-6-methylgeldanamycin, (8) 8-Demethylgeldanamycin or 17-O-Demethylgeldanamycin, (9) Geldanamycin, (10) C_30_H_40_N_2_O_10_ (geldanamycin related but with a molecular formula not found in the Dictionary of Natural Products), (11) Nigericin, and (12) 30-O-Acetylnigericin. Dereplicated components in sample A191: (1) Antibiotic RK 397, (2) Flavofungin I, (3 and 4) – C_36_H_56_O_9_ (related to flavofungin but with a molecular formula not included in the Dictionary of Natural Products), (5) Flavofungin II, (6) Prodigiosin 25b, (7) C_18_H_30_O (molecular formula not present in the Dictionary of Natural Products as produced by prokaryotes), (8) Undecylprodigiosin, (9) Spectinabilin or Arabilin, (10) Lyngbyamide D, (11) C_28_H_33_NO_5_ (molecular formula not found in the Dictionary of Natural Products as produced by prokaryotes), and (12) Pamamycins.

To uncover the biosynthetic abilities of the airborne *Streptomyces* strains, ethyl acetate extracts were screened for secondary metabolites production by LC/UV and LC/HRMS analyses in combination with searches in UV and MS databases or the DNP after generation of a molecular formula of each peak based on HRMS results. Most of the strains displayed complex metabolic profiles revealing their high potential as a source of novel natural products (Supplementary Material 1). As an example, Figure 4 displays UV_210nm_ chromatograms corresponding to samples A-185 and A-191 showing identified compounds.

Among a total of 138 metabolites detected by HPLC/MS in the ethyl acetate extracts of the studied strains (Supplementary Material 1), 100 were identified after metabolite profiling analysis and comparison with natural products databases (some of them only at the family level). Among identified products, 45 were reported to display biological activities as antibiotics (17 antifungal and 13 antibacterial), 29 as cytotoxic/ antitumor agents, 5 as antiviral, 3 as antiparasitic, 3 as immunosupresors, 2 as anti-inflammatory, 1 as neuroprotector, 1 as insecticide, and other compounds with physiological roles in the *Streptomyces* life cycle (Table 3).

**Table 3:** Bioactive compounds produced by atmospheric-derived *Streptomyces* strains and their biological activities.

**Compound**	**Strain**	**Biological activities**	**Identification**
2-Acetamidobenzamide	A-196	Antifungal against phytopathogenic filamentous fungi (Phay et al., 1996)	MS
4,5-Dihydrogeldanamycin	A-185, A-229	Anticancer (Schnur et al., 1995; Wu et al., 2012)	MS
6-Prenyltryptophol	A-206	Cytotoxic (Sánchez López et al., 2003)	MS
8-Demethylgeldanamycin/17-O-Demethylgeldanamycin^*^	A-185, A-229	Moderate cytotoxicity against the human breast cancer cell line (Buchanan et al., 2005)/Unknown	MS
Abierixin	A-229	Antibiotic (David et al., 1985), weak cytotoxicity, antimalarial activity (Supong et al., 2016)	MS
Actinorhodin	A-226	Antibiotic (Wright and Hopwood, 1976)	UV
Aggreceride B	A-203, A-206, A-221	Platelet aggregation inhibitor (Omura et al., 1986)	MS
Albonoursin	A-192	Antibacterial, antitumor in mice (Fukushima et al., 1973)	MS
Alpha-lipomycin	A-186	Antibiotic (Bihlmaier et al., 2006)	MS
Alteramide-derivative	A-201, A-209, A-231	Unknown	UV
Alteramide A	A-211, A-214, A-228	Cytotoxic (Shigemori et al., 1992); antifungal (Moree et al., 2014)	MS
Alteramide B	A-211, A-214, A-228	Antifungal (Moree et al., 2014)	MS
Antibiotic RK 397	A-191	Antibiotic, cytotoxic (Kobinata et al., 1993)	MS
Antibiotic TMC (1A/B or TMC 1F)	A-241	Antibiotic, moderate cytotoxicity (Kohno et al., 1996)	MS
Antimycins (A4; A5a/A5b; A6a/A6b/A18 and A11)	A-206, A-222	Antifungal (Seipke et al., 2012); antiviral (Raveh et al., 2013); cytotoxic (Takimoto et al., 1999); apoptosis inducer (Seipke and Hutchings, 2013)	UV, MS
Bafilomycin C1	A-228	Antibiotic, cytotoxic (Moon et al., 2003)	MS
Blastmycin	A-222	Fungicide (Endo and Yonehara, 1970), cytotoxic (Fujita et al., 2004)	MS
Caboxamycin	A-228	Anti-Gram-positive, antitumor (Hohmann et al., 2009)	UV
Cyclo(4-hydroxyprolylleucyl)	A-193	Moderate toxicity toward brine shrimp larvae (Gao et al., 2014)	MS
Cyclo(leucylprolyl)	Several strains^A^	Antibiotic, cytotoxic activity (Santos et al., 2015)	MS
Cyclo(prolylvalyl)	A-197, A-203, A-221, A-225, A-241	Antifungal (Kumar et al., 2014)	MS
Deisovalerylblastmycin	A-222	Antifungal (Ishiyama et al., 1976)	MS
Dihydromaltophilin	A-209	Antifungal (Fiedler et al., 2005)	MS
Feigrisolide C	A-214	Moderate activity on Coxsackie virus B3 (Tang et al., 2000), lysis of *Plasmopara viticola, Phytophthora capsici*, and *Aphanomyces cochlioides* zoospores (Islam et al., 2016)	MS
Flavofungin I and II	A-191	Antifungal antibiotic (Uri and Bekesi, 1958), anti-glioma and antifungal activities (Wang et al., 2017a)	MS
Fogacin	A-226	Antimicrobial activities against *C. albicans* (Lu et al., 2014)	MS
Geldanamycin	A-185; A-229	Antifungal, anticancer, neurotrophic and neuroprotective (Tadtong et al., 2007)	MS
Germicidin A	Several strains^B^	Spore germination, hypha elongation (Aoki et al., 2011)	MS
Germicidin B	A-193, A-203, A-217, A-221, A-226	Spore germination, hypha elongation (Aoki et al., 2011)	MS
Germicidin D	A-193	Spore germination, hypha elongation (Aoki et al., 2011)	MS
Grecocycline A	A-202	Cytotoxic (Paululat et al., 2010)	MS
Griseorhodins	A-214	Antibiotics, cytotoxic (Stroshane et al., 1979); inhibition of HIV reverse transcriptase and human telomerase (Lin et al., 2014)	UV
Herbimycin E	A-185	Hsp90α affinity (Alzheimer's disease pathogenesis) (Shaaban et al., 2013)	MS
Ikarugamycin epoxide	A-214	Moderate activities against Gram-positive bacteria and fungi, strongly cytotoxic (HMO2 and MCF 7) (Bertasso et al., 2003)	MS
Ilamycin A, C1 or C2	A-215	Cytotoxic (Ma et al., 2017)	MS
Indanomycin	A-222	Antibacterial, insecticidal (Zhang et al., 1997)	MS
Izumiphenazine C	A-196	Synergistic activity in sensitizing TRAIL-resistant AGS cells (Abdelfattah et al., 2010)	MS
Juglomycin A	A-215	Antibiotic (Fiedler et al., 1994)	MS
Kandenol C	A-228	Moderate antimicrobial activity againts *Mycobacterium vaccae* (Ding et al., 2012)	MS
Ketomycin	A-185	Antibiotic (Takeda et al., 1984)	MS
Lobophorin A	A-204, A-217	Anti-inflammatory, antituberculosis, anti-BCG (Jiang et al., 1999; Chen et al., 2013)	UV, MS
Lobophorin B	A-204, A-218	Anti-inflammatory, antituberculosis, anti-BCG (Jiang et al., 1999; Chen et al., 2013)	UV, MS
Lobophorin K	A-204, A-219	Cytotoxic, moderate antibiotic activity againts *Staphylococcus aureus* (Braña et al., 2017a)	UV, MS
Maltophilins	A-214, A-228	Antifungal (Fiedler et al., 2005)	UV, MS
Methylsulfomycin I	A-209	Antibiotic (Vijaya Kumar et al., 1999)	MS
N-Butanoylhomoserine lactone	A-228	Quorum-sensing signal molecule in Gram-negative bacteria (Chan et al., 2011)	MS
Neoenactin B1 or B2	A-221	Antifungal (Roy et al., 1987)	MS
Nigericin	A-185, A-229	Antibiotic, strong cytotoxicity (A2780 and SKOV3) (Wang et al., 2017b)	MS
Nonactins	A-209, A-214	Ammonium ionophore, antibacterial, antiviral, antitumor (Zhan and Zheng, 2016)	MS
Okicenone	A-189	Cytotoxic activity (Komiyama et al., 1991)	MS
Oxostaurosporine	A-198	Protein kinase C inhibitor (Osada et al., 1992)	MS
Pamamycins (607, 621 A/B/C/D, 635 A/B/C/D/E/F and 663)	A-191	Aerial mycelium and secondary metabolite production inducing (Hashimoto et al., 2011), anti-Gram-positive and antifungal (Hanquet et al., 2016)	MS
Paulomycin B	A-231	Anti-Gram-positive, gonococcal and *Chlamydia* infections (Argoudelis et al., 1982; Novak, 1988)	UV
Phenazinoline or Izumiphenazine derivative	A-196	Unknown	MS
Phenelfamycin	A-189, A-210	Anti-Gram-positive (Brötz et al., 2011)	UV
Radamycin	A-209	tipA promoter inducer (González Holgado et al., 2002)	MS
Reblastatin	A-185	Cell cycle inhibitor (Takatsu et al., 2000), Hsp90 ATPase inhibitor (Wu et al., 2012)	MS
Spectinabilin/Arabilin^*^	A-191	Antimalarial and cytotoxic (Isaka et al., 2002)/Androgen receptor antagonist in prostate cancer LNCaP cells (Kawamura et al., 2010)	MS
Staurosporine	A-198, A-230	Protein kinase C inhibitor (Mori et al., 1994), Antifungal, pan-kinase inhibitor (Tamaoki et al., 1986; Song et al., 2017)	MS
Tetranactin	A-214	Antibiotic, immunosuppressive and anti-proliferative (Tanouchi and Shichi, 1988)	MS
Thiazinogeldanamycin	A-185	Cytotoxic (Ni et al., 2011)	MS
Trinactin	A-214	Antibiotic, immunosuppressive (Tanouchi and Shichi, 1987)	MS
Trioxacarcin A	A-186	Antitumor, antibiotic (Tomita et al., 1981)	MS
Undecylprodigiosin	A-191, A-193, A-203, A-226, A-241	Antibiotic, cytotoxic (Petrović et al., 2017), immunosuppressor (Songia et al., 1997; Williamson et al., 2006)	UV, MS
Urauchimycin A/B and C	A-222	Antibiotic (Imamura et al., 1993)	MS
Valinomycin	A-211	Antibiotic, antiparasitary, antiviral (Perkins et al., 1990; Cheng, 2006; Pimentel-Elardo et al., 2010)	MS
WS 9326A	A-198	Tachykinin receptor antagonist (Hayashi et al., 1992); quorum sensing inhibitor in Gram-positive bacteria (Desouky et al., 2015)	MS
β-Indomycinone/Saptomycin A/Rubimycinone A^*^	A-197	Antibiotic, cytotoxic (Tsukahara et al., 2014)/Antimicrobial (Abe et al., 1993)/Unknown	MS

*The asterisk means that more than one compound was identified*.

*A: A-189, A-197, A-201, A-203, A-206, A-214, A-221, A-222, A-225, A-228, A-230*.

*B: A-186, A-193, A-196, A-203, A-204, A-217, A-221, A-222, A-226*.

Remarkably, there are 38 metabolites whose molecular formulae determined by HRMS does not correspond to any compound included in Natural Products Databases and remained unidentified. These molecules might be new natural products, and therefore constitute an excellent starting point for the discovery of new bioactive molecules with pharmaceutical interest.

## Discussion

We provide here the first insight ever made into the *Streptomyces* species diversity within a small storm cloud sample, which was collected after a hailstone and rainwater precipitation event during the afternoon of the 14th of February 2016 in the Cantabrian Sea coast (North of Spain). Our results revealed a striking richness of *Streptomyces* species present in the atmosphere. A generalized feature observed in the airborne strains here isolated (with a single exception) is their high halotolerance, since they mostly grew very well in culture media containing 7% NaCl, thus suggesting a marine origin. This has been previously proposed for *Streptomyces* species isolated from atmospheric precipitations in Northern Spain (Braña et al., 2015; Sarmiento-Vizcaíno et al., 2016).

Air mass backward trajectories analysis of this precipitation event revealed two main sources, oceanic (mainly from the Arctic Ocean, Greenland and Iceland) and continental (Canada) depending on the altitude. Mixing of the different air layers during the travel was observed. All air masses crossed the Atlantic Ocean and arrived to continental Europe after 4 days, reaching the North of Spain where samples were collected by atmospheric precipitation. Consistent with estimated air mass backward trajectories all isolated strains are similar to previously isolated species from highly diverse environments, either marine or terrestrial, mainly from the North hemisphere. The results of Blast search from 16S rRNA partial sequences herein provided revealed the presence of 29 strains belonging to 20–25 different *Streptomyces* species. Bearing in mind that the currently estimated number of *Streptomyces* species is of 550–823, the number of species isolated during this hail event represents a non-negligible 3–4% of all *Streptomyces* species known so far in our planet. This fact suggests that the presence in the atmosphere could be a generalized phenomenon within the *Streptomyces* genus and is in agreement with our previously stablished atmospheric dispersion model (Sarmiento-Vizcaíno et al., 2016). This model has recently received further support from culture-independent report from precipitations in Japan. That work also shows seasonal variations of microbial communities in the atmosphere in correlation with estimated air mass trajectories (Hiraoka et al., 2017).

Overall, the most relevant feature of the atmospheric-derived *Streptomyces* strains here studied is that they represent a striking great reservoir of structurally diverse bioactive compounds (Table 3). One hundred molecules have been identified, and for 60 of them different biological activities, mainly antimicrobial (antibacterial, antifungal, and antiviral) and antitumor properties, have been previously described. Interestingly 38 potentially bioactive natural products have not been identified and their possible novelty is the subject of current active research. During the time of writing this manuscript a new natural product was identified, after purification and NMR structure elucidation, in one of the strains here isolated (unpublished results). The number of produced secondary metabolites for these strains is estimated to be much higher than the one presented here, since only diffusible apolar molecules produced in a unique culture condition (R5A medium, 28°C) were analyzed so far, and possible diffusible polar or volatile products were not analyzed. Even more, most of the *Streptomyces* metabolic abilities are mainly hidden, not expressed under standard culture conditions. This silent (or cryptic) potential represents most of the metabolome (Reen et al., 2015).

## Conclusion

Our findings highlight the relevance of the atmosphere as a novel source of highly diverse *Streptomyces* species able to produce an incredible reservoir of natural products, which has been overlooked so far. Atmospheric precipitations might represent a relevant unexplored environment for discovering bioactive natural products with pharmacological and biotechnological interest.

## Author contributions

GB isolated the strains and analyzed the air masses backward trajectories. AS-V performed the taxonomic identification and phylogenetic analyses of the strains. AS-V and JE conducted the bioactivity assays. AS-V and AB analyzed the compounds by LC-UV. JM and FR performed the metabolite profiling analysis and identified the compounds produced by LC-MS. GB wrote the manuscript which has been revised and approved by all the authors. LG and GB conceived and coordinated the project.

### Conflict of interest statement

The authors declare that the research was conducted in the absence of any commercial or financial relationships that could be construed as a potential conflict of interest.

## Supplementary material

Supplementary Material 1 UV 210 nm chromatograms corresponding to all samples.
