# Supplementary figures and images for: Atmospheric Precipitations, Hailstone and Rainwater, as a Novel Source of Streptomyces Producing Bioactive Natural Products
Source: Front Microbiol. 2018 Apr 23;9:773. doi: 10.3389/fmicb.2018.00773 (PMC5924784; doi:10.3389/fmicb.2018.00773)

A185

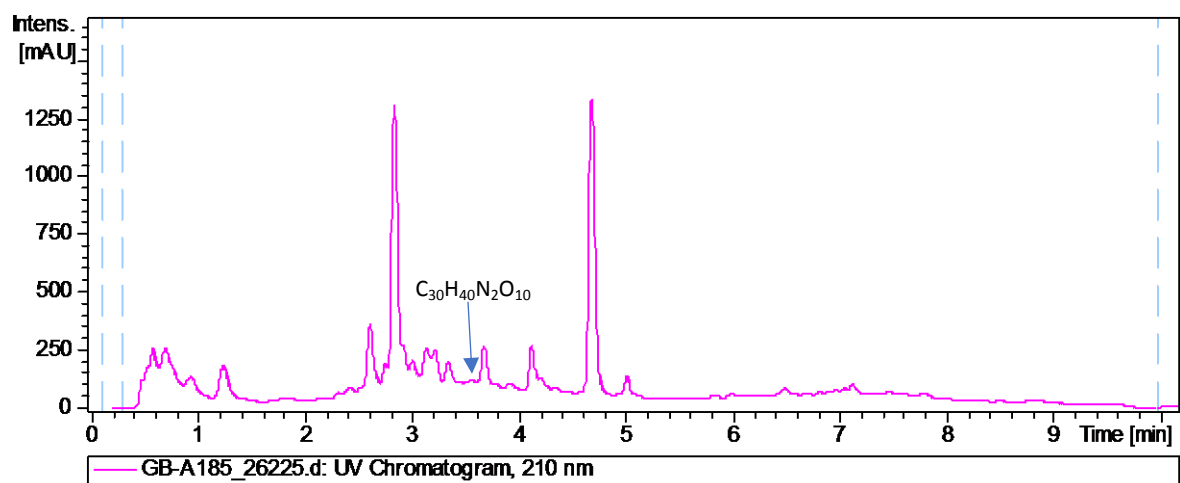

A189

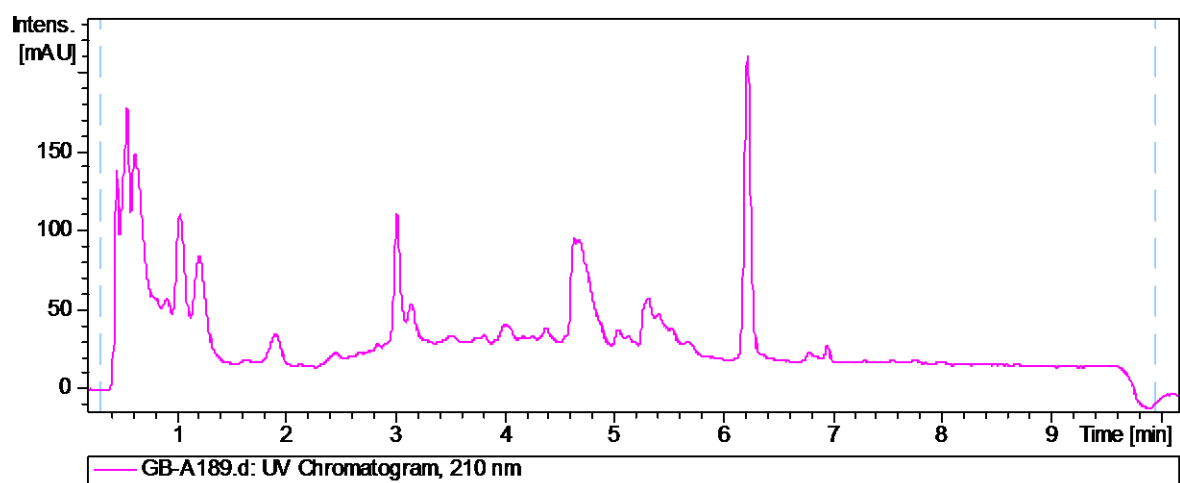

A191

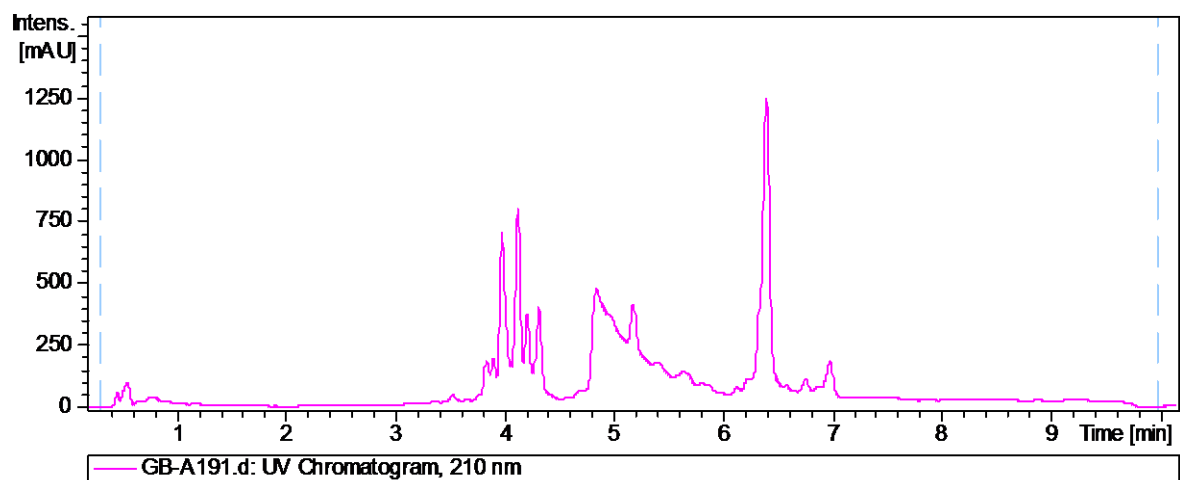

A192

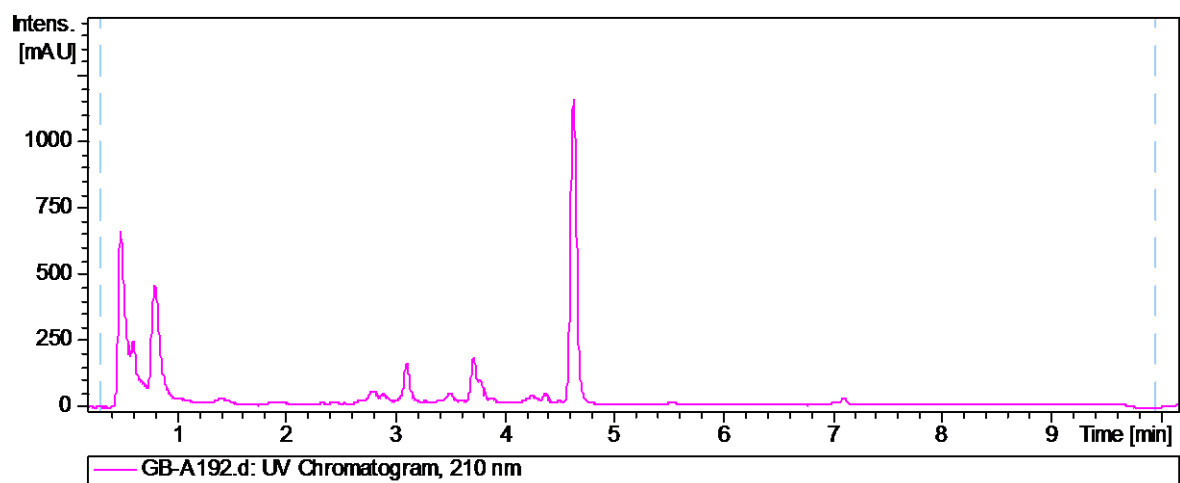

A193

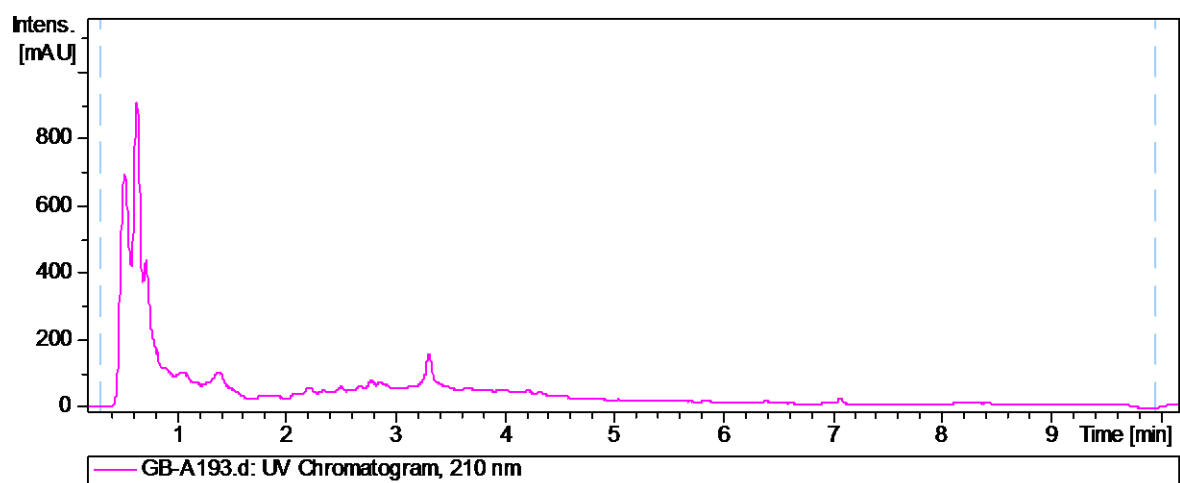

A196

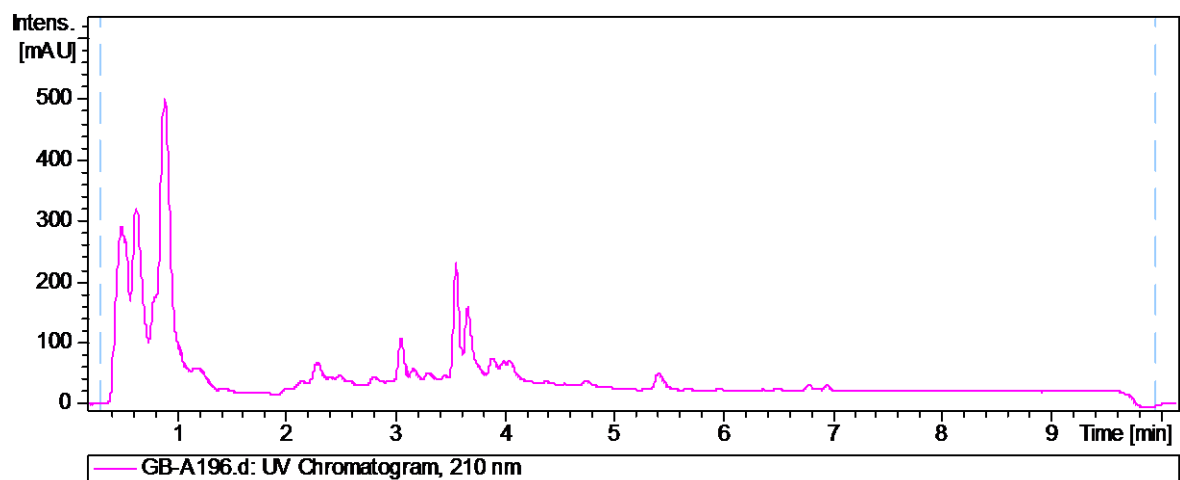

A197

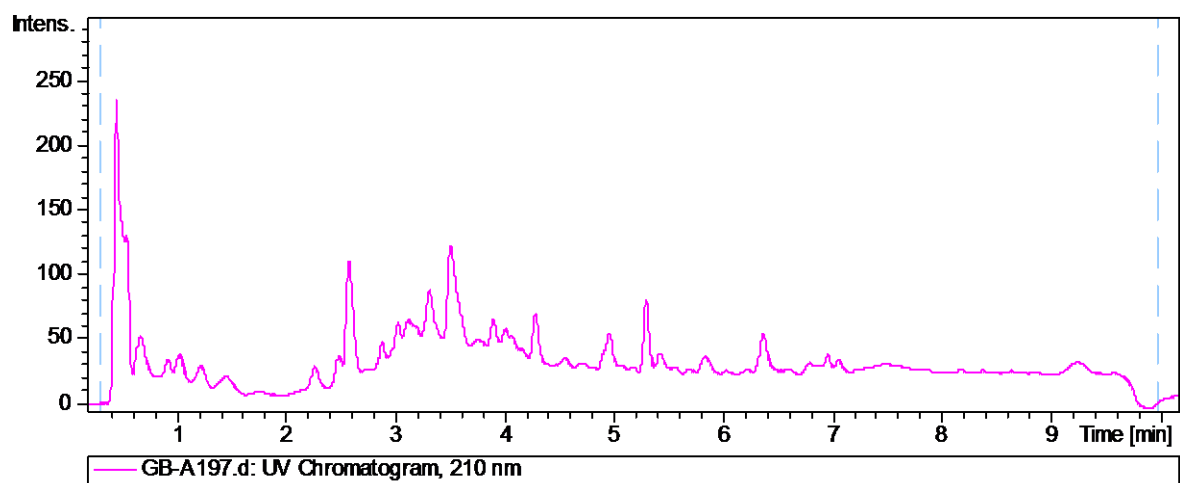

A198

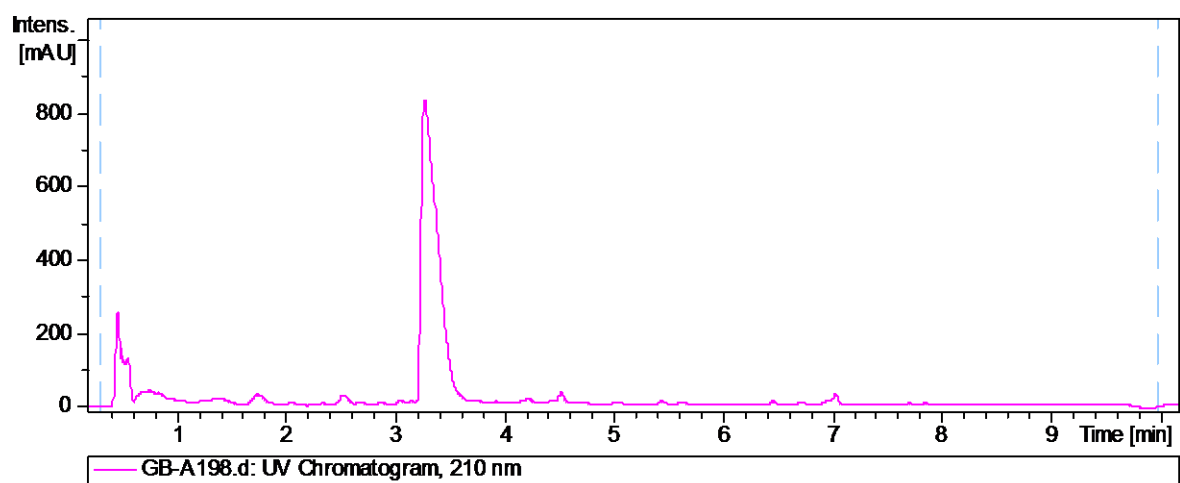

A201

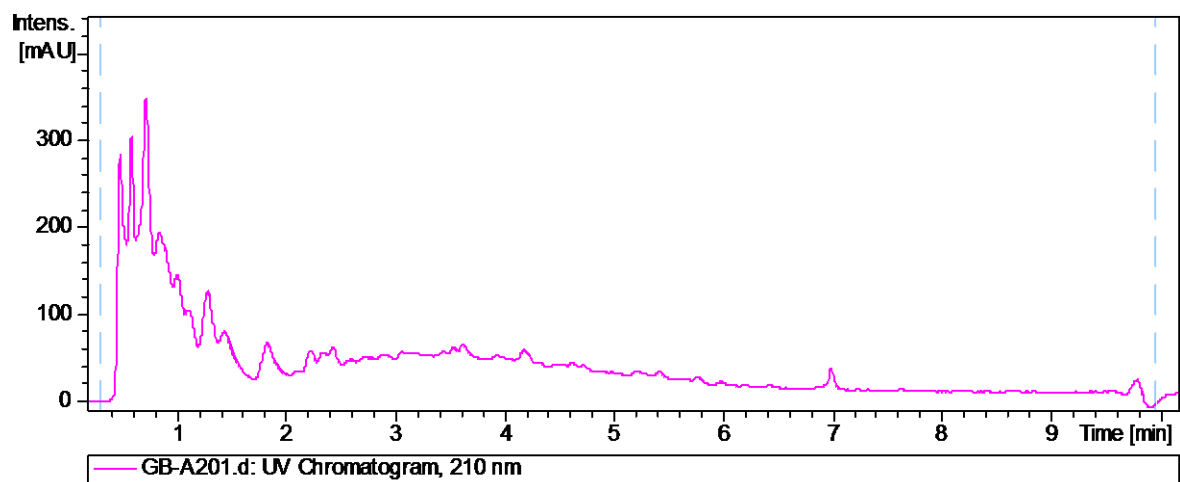

A203

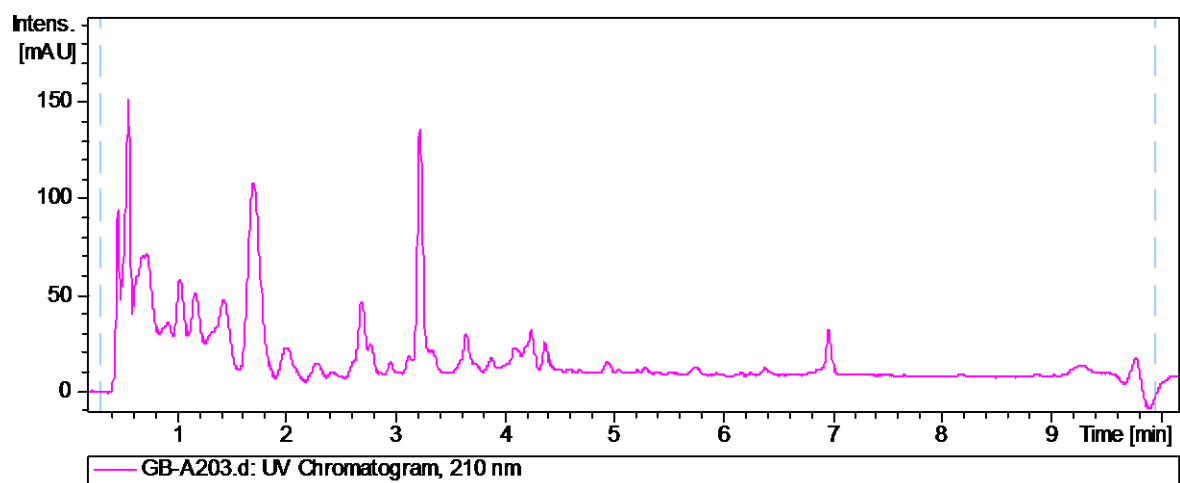

A206

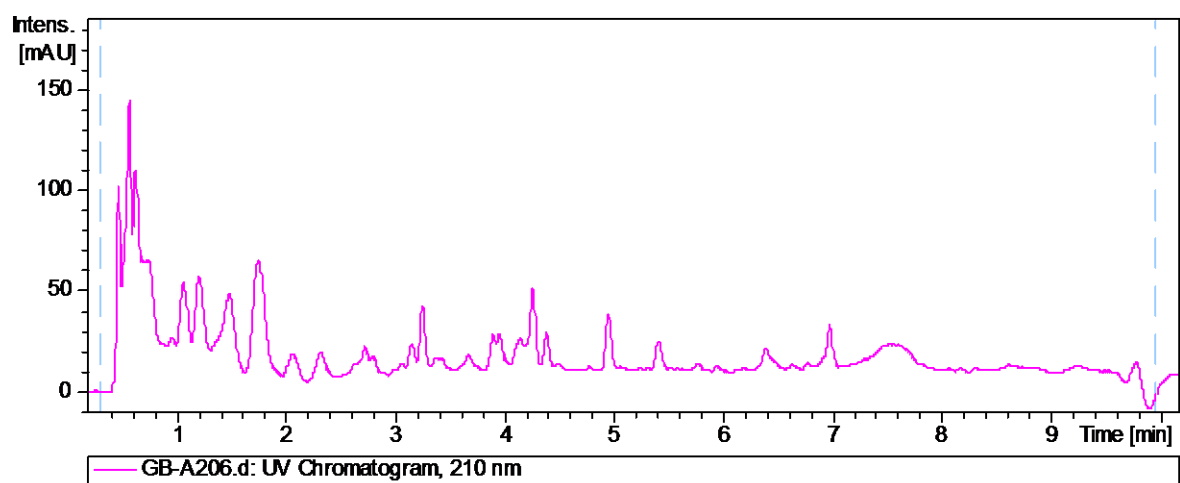

A211

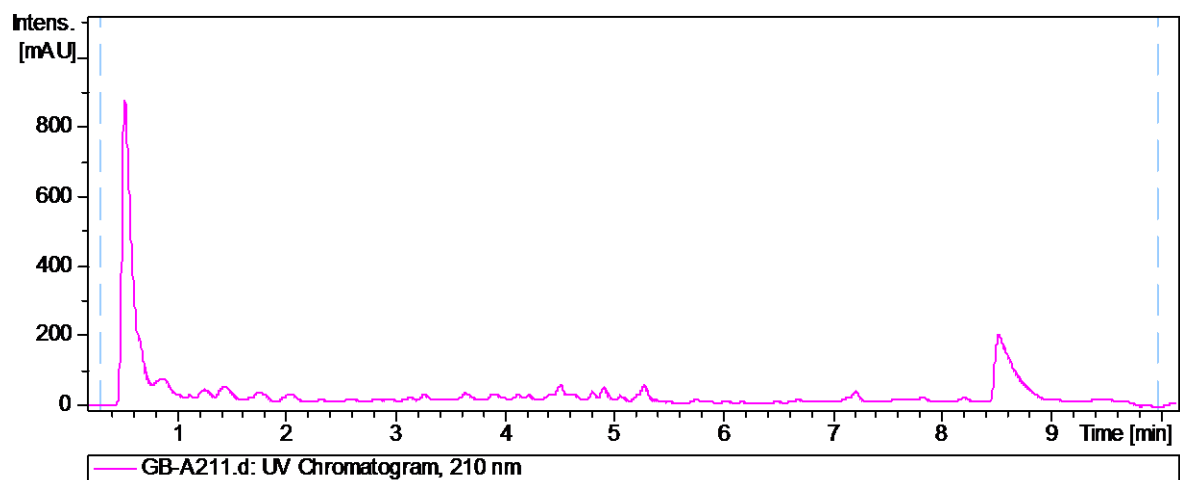

A214

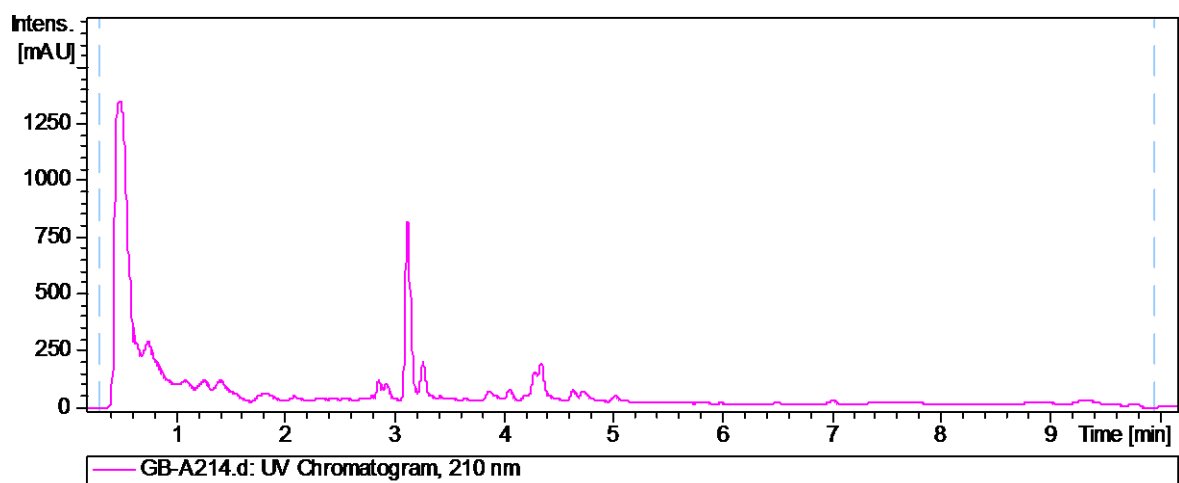

A221

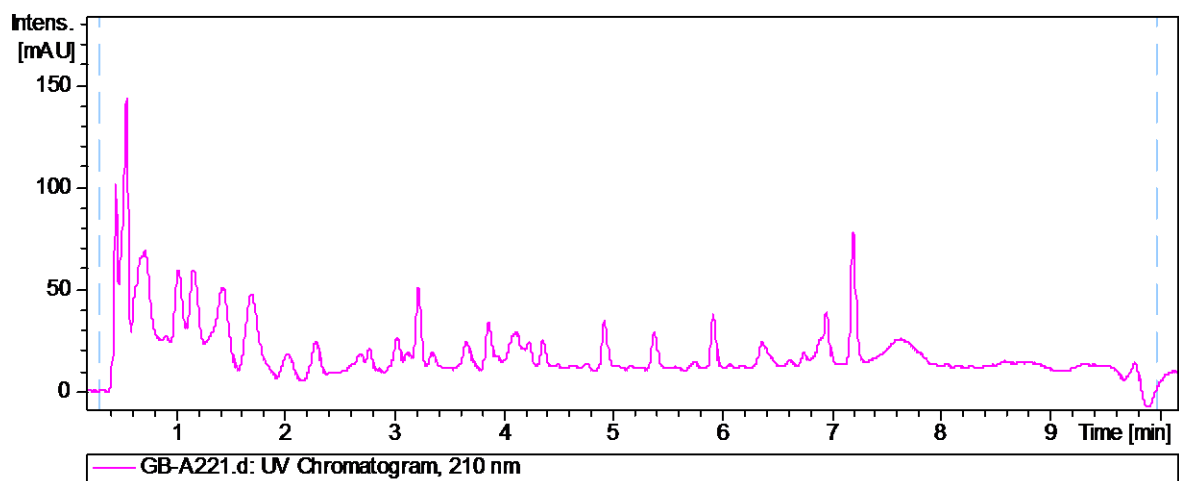

A222

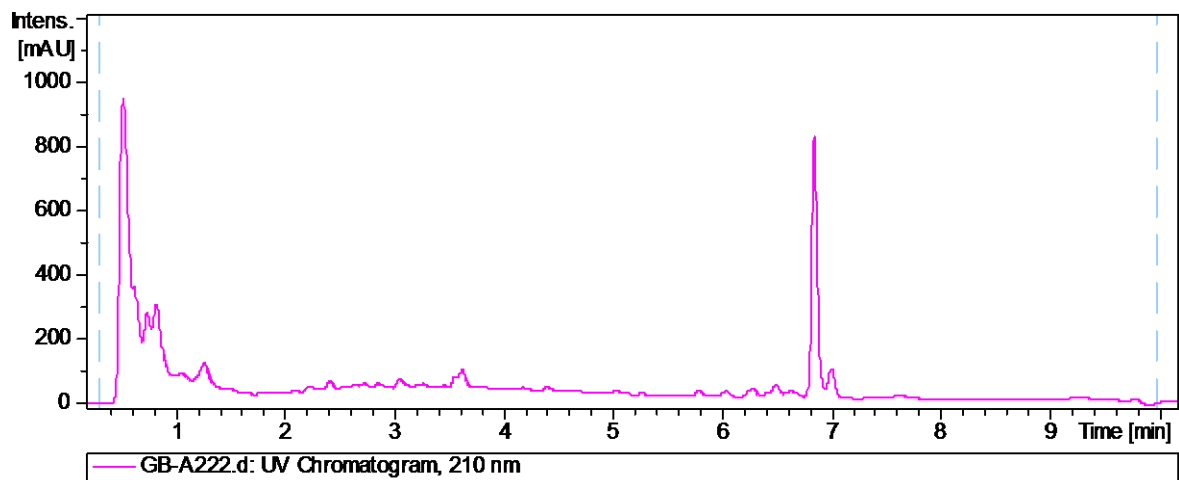

A225

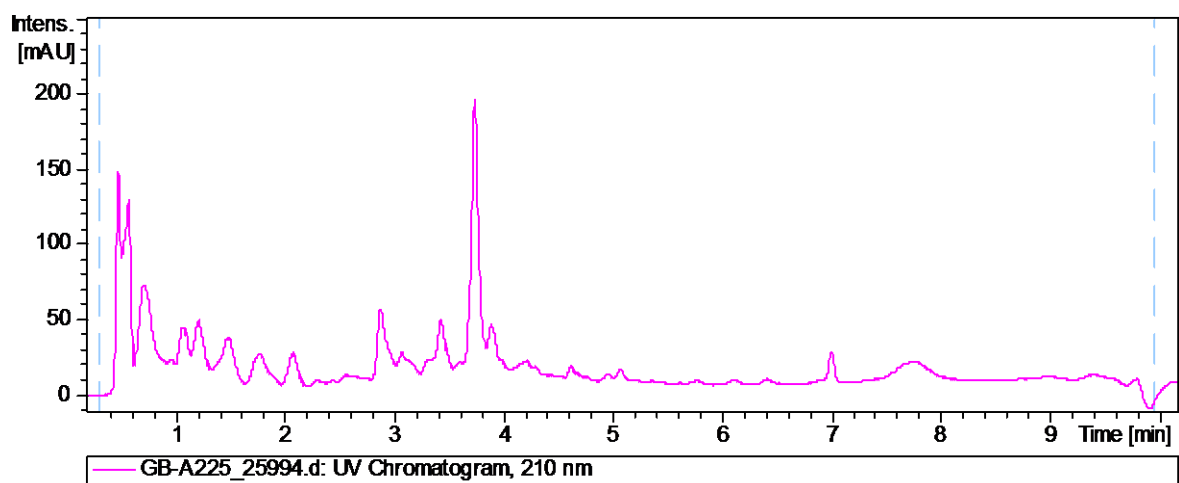

A226

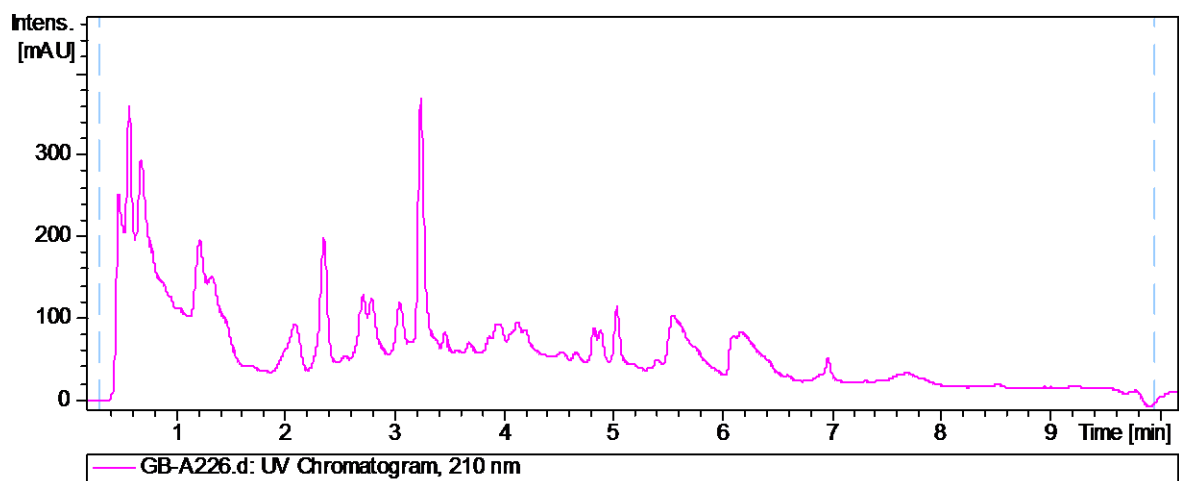

A228

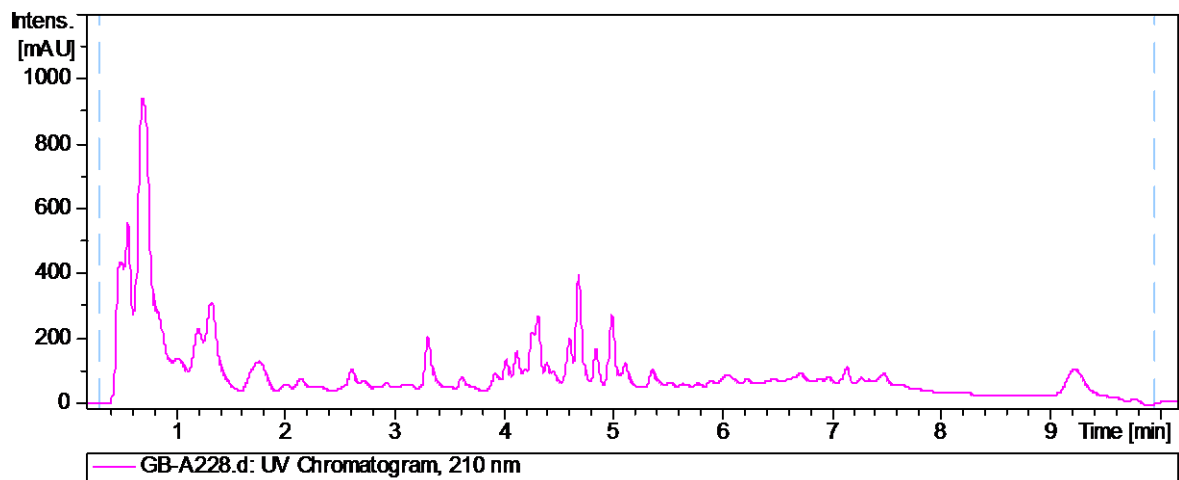

A229

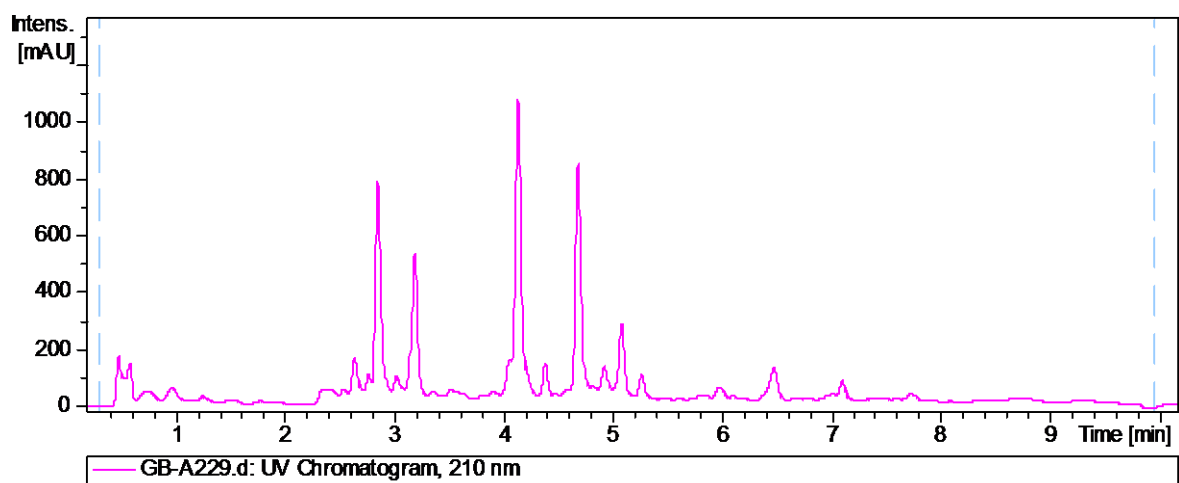

A230

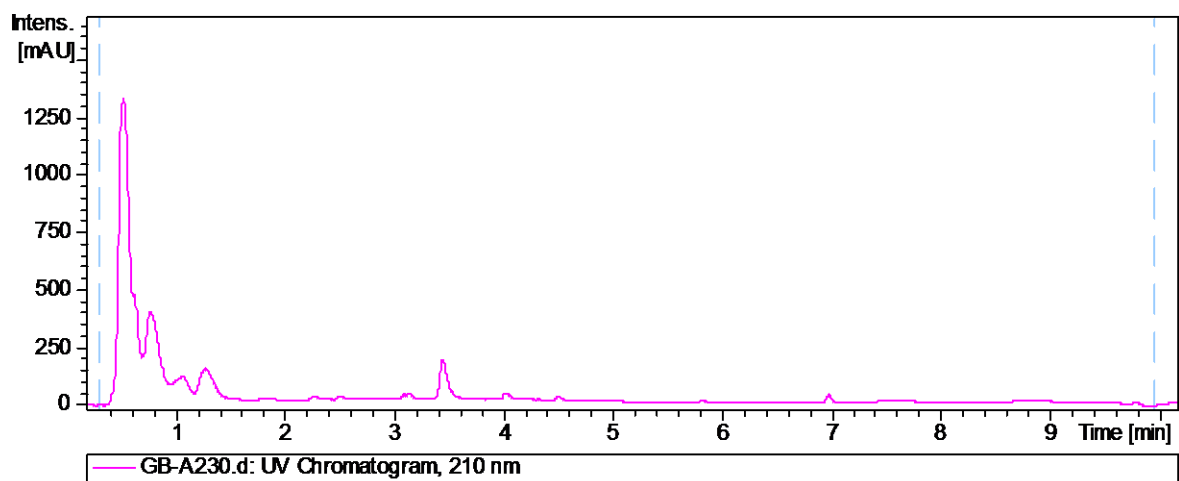

A241

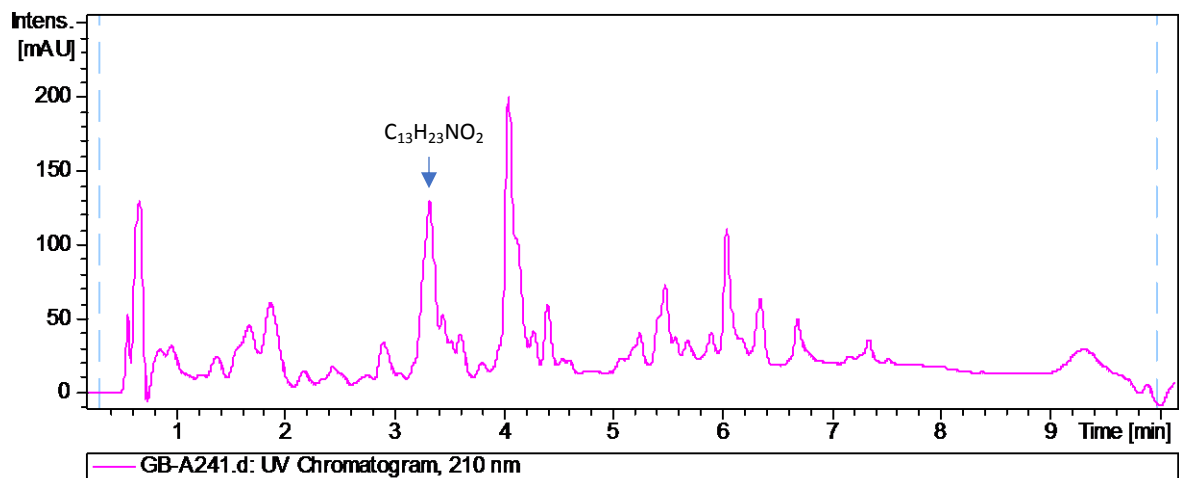

Supplement: Supplementary Material 1 — UV 210 nm chromatograms corresponding to all samples. [file Image1.PDF]
